# Global, regional, and national burden of ischemic heart disease associated with diet high in sodium from 1990 to 2021, and its projections to 2035: a systematic analysis of the global burden of disease study 2021

**DOI:** 10.3389/fnut.2025.1630331

**Published:** 2025-09-16

**Authors:** Yuqin Mao, Jiong Wang, Shaoyan Xuan, Minxiu Wang, Shu Yang, Qiuji Wu, Zhihua Tang

**Affiliations:** ^1^Department of Pharmacy, Shaoxing People’s Hospital, Shaoxing, China; ^2^School of Medicine, Shaoxing University, Shaoxing, Zhejiang, China; ^3^State Key Laboratory of Genetic Engineering, Department of Pharmaceutical Sciences, School of Life Sciences, Fudan University, Shanghai, China; ^4^MOE Engineering Research Center of Gene Technology, School of Life Sciences, Fudan University, Shanghai, China

**Keywords:** ischemic heart disease, global burden of disease, diet high in sodium, risk factors, health projections

## Abstract

Numerous studies have demonstrated an association between high-sodium diet and the incidence of ischemic heart disease (IHD). This study aims to assess the global impact of IHD associated with diet high in sodium from 1990 to 2021. We retrieved relevant data from the Global Burden of Disease Study 2021 (GBD 2021). The data encompassed the number of IHD-related deaths due to high-sodium intake, age-standardized mortality rates (ASMR), disability-adjusted life years (DALYs), and age-standardized DALYs rates (ASDR), all of which were estimated using GBD’s statistical model. Subsequently, we calculated the burden of IHD associated with high-sodium intake for each country and territory, stratified by age, sex, and socio-demographic index (SDI). The Bayesian age-period-cohort (BAPC) model was used to predict future trends of the IHD burden up to 2035. We discovered that excessive dietary sodium intake is associated with an elevated global burden of IHD. Although ASMR and ASDR have declined over time, this situation continues to impede global socioeconomic progress, and this trend might be associated with population growth and aging. These paradoxical trends underscore the urgent necessity for public health policymakers to prioritize the development of sodium reduction strategies customized to regional epidemiological patterns and gender-specific risk profiles.

## Introduction

Ischemic heart disease (IHD) is one of the leading causes of high global incidence and mortality rates and represents the most burdensome type of cardiovascular disease (CVD) (1). It is a complex disease characterized by coronary artery stenosis, which results in myocardial ischemia, hypoxia, and even necrosis. In 2019 alone, IHD caused 9.14 million deaths (2, 3). The burden of IHD on sustainable development in the 21st century is significant. Despite advancements in treatment strategies and preventive measures, IHD remains a major public health challenge for countries and regions (4). The 2019 Global Burden of Disease (GBD) study estimated that there were approximately 197 million cases of IHD worldwide in 2019 (5). It is noteworthy that the attributable burden data reported in the GBD are derived from a statistical synthesis of surveys, hospital records, death certificates, and literature-based relative risks, rather than direct counts.

The influence of dietary risk factors on CVD has received attention, and their importance as a major factor was widely acknowledged (6). High-sodium diet has emerged as a significant risk factor associated with various health complications, and mounting evidence indicates a significant association between excessive sodium intake and the onset and progression of CVD (7, 8). As per the recommendations of the World Health Organization (WHO), the daily dietary sodium intake of healthy adults should be limited to less than 2 g [WHO Sodium Reduction Fact Sheet (2025)] (9). Existing studies have demonstrated that diet high in sodium (DHIS) is associated with elevated blood pressure and may be a significant contributing factor to the increased risk of CVD-related mortality in adults (10). Therefore, the high-sodium diet may substantially elevate the risk of developing IHD.

In this study, we conducted a comprehensive analysis of the GBD database to assess the global burden of IHD associated with diet high in sodium from 1990 to 2021. This study aims to establish an evidence-based framework for policymakers and public health stakeholders to formulate targeted interventions, offering novel perspectives for optimizing prevention strategies and therapeutic approaches in IHD management.

## Materials and methods

### Study data

This study utilized data on ischemic heart disease mortality and DALYs associated with diet high in sodium from 1990 to 2021, obtained from the Global Health Data Exchange (GHDx) query tool,^1^ based on gender, age, region, and country. This study classified 204 countries and territories into five distinct levels according to their Socio-Demographic Index (SDI) as defined by GBD: high, high-middle, middle, low-middle, and low SDI. We calculated the SDl through several social factors, with the fertility rate of the population aged <25 years, the education level of the population aged >15 years, and per capita income. Based on geographic continuity, the world was divided geographically into 21 distinct GBD regions, such as Western Europe and East Asia. Utilizing the latest epidemiological data and refined standardized methods, the GBD 2021 study provides a comprehensive assessment of health loss associated with 371 diseases, injuries, and impairments, as well as 87 risk factors in 204 nations and territories (11).

This study is based on data from the GBD 2021 study and employs a comparative risk assessment (CRA) framework to estimate the attributable burden of ischemic heart disease (IHD) due to diet high in sodium. The definition of IHD employed in this study adheres to the International Classification of Diseases (ICD). It encompassed corresponding diagnostic codes within this classification: I20-I21.6, I21.9- I25.9 (11). Diet high in sodium is defined as a daily sodium intake exceeding the theoretical minimum risk exposure level (TMREL), as an average 24-h urinary sodium excretion of 1–5 g/day. This study is from multiple data sources, including dietary surveys, urinary sodium monitoring, and relevant epidemiological studies, were integrated to estimate sodium intake exposure distributions across regions, ages, and genders. A spatio-temporal gaussian process regression (ST-GPR) model is used for spatial and temporal estimation. The population attributable fraction (PAF) is used to calculate the burden of IHD associated with diet high in sodium exposure above the TMREL.

### Statistical analysis

The burden of ischemic heart disease associated with diet high in sodium was assessed using metrics including numbers of mortality and DALYs, the age-standardized mortality rate (ASMR), age-standardized DALYs rate (ASDR). DALYs were computed as the sum of the years lived with disability and the years of life lost. According to the age group construction of the standard population, the ASR (per 100, 000 population) were calculated using the following formula:


$$\mathrm{ASR}=\frac{\sum_{i=1}^{A}a_{i}\,w_{i}}{\sum_{i=1}^{A}a_{i}}\times 100,000,$$

(a_*i*_ refers to the incidence of the ith age group. w_*i*_ denotes the number of persons (or weight) in the same age subgroup i of the assigned reference standard population).

Estimated annual percentage change (EAPC) is used to estimate the trends of the ASRs, and it quantitatively calculates the average annual rate of change of ASR for a specified period (12), which follows this formula, i.e., *y* = α + β*x* + ε, where *y* = ln (ASR), and *x* = the calendar year. The EAPC calculation formula, 100 × (exp(β) −1), and its 95% confidence intervals (CI) can also be calculated from the linear regression model.

In this study, we employed a Bayesian age–period–cohort (BAPC) modeling framework to estimate and project temporal trends in disease burden. Consistent with the classical APC approach, temporal variation was decomposed into additive contributions from age, period, and birth cohort effects. To ensure smoothness of the estimated effects and enhance predictive performance, we specified second-order random walk (RW2) priors for each temporal dimension, under the assumption that changes between adjacent points are gradual and temporally correlated. Parameter estimation was conducted using integrated nested Laplace approximation (INLA). The model rests on the following key assumptions: (1) age, period, and cohort effects evolve smoothly over time; (2) the effects are additive and independent; and (3) observed historical patterns can be extrapolated into the projection period in the absence of major unforeseen shocks (13, 14).

All data analyses were performed using the open-source software R (version 4.3.3). Statistical significance was set at *P* < 0.05.

## Results

### The burden of ischemic heart disease associated with diet high in sodium at global level

Between 1990 and 2021, the global mortality cases of ischemic heart disease associated with diet high in sodium increased by 81%, rising from 364725 (95% uncertainty interval (UI): 73128–895398) to 658807 (95% UI: 116243–1564791). The age-standardized mortality rate (ASMR) of IHD associated with diet high in sodium decreased by 24%, dropping from 10.38 per 100 000 population (95% UI: 1.91–26.03) in 1990 to 7.86 per 100 000 population (95% UI: 1.35–18.78) in 2021, with an estimated annual percentage change (EAPC) of −0.91 (95% confidence interval (CI): −0.96 to −0.86) (Table 1).

**TABLE 1:** Mortality cases and ASMR of IHD in 1990 and 2021, and temporal trends.

	1990		2021		1990–2021 EAPC of ASMR (95% CI)
	Mortality cases, No. ×10^3^	ASMR per 100,000 (95% UI)	Mortality cases, No. ×10^3^	ASMR per 100,000 (95% UI)	
Global	364725 (73128, 895398)	10.38 (1.91, 26.03)	658807 (116243, 1564791)	7.86 (1.35, 18.78)	−0.91 (−0.96, −0.86)
**Socio-demographic index**					
High SDI	89875 (11264, 258934)	8.08 (1.00, 23.39)	83947 (9255, 223993)	3.57 (0.41, 9.48)	−2.78 (−2.86, −2.69)
High-middle SDI	128962 (31506, 290104)	14.66 (3.33, 33.85)	209156 (45969, 464877)	10.71 (2.32, 23.91)	−1.15 (−1.30, −1.00)
Middle SDI	95255 (24567, 202693)	11.13 (2.48, 24.42)	243557 (51321, 543375)	9.94 (1.91, 22.53)	−0.22 (−0.32, −0.13)
Low-middle SDI	40146 (4943, 107895)	7.49 (0.83, 20.71)	99973 (9064, 276250)	7.63 (0.64, 21.46)	0.19 (0.12, 0.26)
Low SDI	9406 (403, 29168)	4.97 (0.21, 15.60)	21229 (691, 65223)	5.09 (0.15, 15.89)	0.23 (0.14, 0.33)
**Region**					
Andean Latin America	1038 (40, 2842)	5.84 (0.21, 16.05)	1962 (61, 5606)	3.49 (0.11, 10.01)	−1.91 (−2.32, −1.50)
Australasia	918 (3, 4131)	4.00 (0.01, 17.98)	633 (2, 2728)	1.08 (0.00, 4.54)	−4.47 (−4.59, −4.35)
Caribbean	1991 (20, 6510)	8.72 (0.09, 28.09)	2472 (19, 8247)	4.51 (0.03, 15.12)	−2.29 (−2.51, −2.08)
Central Asia	12278 (1699, 27997)	30.20 (4.11, 68.68)	11295 (711, 29637)	17.96 (1.12, 46.05)	−2.17 (−2.37, −1.97)
Central Europe	57016 (19608, 103965)	42.12 (13.98, 77.42)	47761 (13167, 91651)	20.07 (5.54, 38.58)	−2.70 (−2.82, −2.59)
Central Latin America	6555 (760, 16319)	9.31 (1.03, 23.22)	17392 (1846, 45097)	7.30 (0.76, 18.97)	−0.97 (−1.18, −0.75)
Central Sub-Saharan Africa	373 (0, 2006)	2.37 (0.00, 12.13)	919 (0, 4347)	2.44 (0.00, 10.90)	−0.15 (−0.23, −0.06)
East Asia	87596 (30715, 158386)	12.71 (3.77, 24.35)	265266 (75668, 521847)	13.36 (3.44, 27.06)	0.54 (0.28, 0.80)
Eastern Europe	36516 (1818, 114542)	14.26 (0.65, 45.62)	40062 (1609, 129822)	11.22 (0.50, 36.29)	−1.26 (−1.76, −0.76)
Eastern Sub-Saharan Africa	3481 (334, 8290)	5.78 (0.54, 13.90)	6364 (358, 16676)	4.96 (0.28, 12.76)	−0.75 (−0.85, −0.66)
High-income Asia Pacific	16007 (3974, 31607)	8.79 (2.06, 17.67)	14484 (1784, 33923)	2.46 (0.32, 5.69)	−4.39 (−4.59, −4.19)
High-income North America	20516 (63, 78434)	5.64 (0.02, 21.54)	23399 (450, 76327)	3.39 (0.07, 10.95)	−1.86 (−2.02, −1.70)
North Africa and Middle East	5474 (2, 28051)	3.60 (0.00, 19.47)	10534 (5, 55165)	2.57 (0.00, 14.10)	−1.20 (−1.25, −1.15)
Oceania	309 (45, 749)	14.85 (2.04, 34.75)	797 (92, 1932)	13.90 (1.61, 32.93)	−0.34 (−0.42, −0.26)
South Asia	33783 (1287, 99568)	6.45 (0.20, 19.50)	106546 (5897, 304733)	7.73 (0.37, 22.62)	0.87 (0.74, 1.00)
Southeast Asia	32815 (9111, 64793)	14.63 (3.70, 29.81)	68034 (12876, 142689)	11.51 (2.00, 24.64)	−0.91 (−0.96, −0.85)
Southern Latin America	3938 (111, 11147)	9.37 (0.26, 26.46)	3127 (90, 8743)	3.45 (0.10, 9.62)	−2.90 (−3.07, −2.74)
Southern Sub-Saharan Africa	485 (1, 1853)	1.98 (0.00, 7.81)	882 (1, 3816)	1.80 (0.00, 7.85)	−0.35 (−0.70, 0.01)
Tropical Latin America	7313 (402, 18994)	9.38 (0.50, 24.54)	10292 (454, 28429)	4.09 (0.18, 11.32)	−2.63 (−2.73, −2.53)
Western Europe	34098 (659, 117210)	5.77 (0.12, 19.77)	21287 (360, 72413)	1.93 (0.04, 6.42)	−3.66 (−3.77, −3.55)
Western Sub-Saharan Africa	2225 (3, 8667)	3.14 (0.00, 12.15)	5300 (14, 19264)	3.54 (0.01, 12.92)	0.52 (0.38, 0.65)

ASMR, age-standardized mortality rate; EAPC, estimated annual percentage change; CI, confidence interval; SDI, socio-demographic index; UI, uncertainty interval.

Similarly, disability-adjusted life years (DALYs) associated with diet high in sodium for IHD increased by 70% between 1990 and 2021, rising from 8296172 (95% UI: 1864218–19526026) to 14079694 (95% UI: 2844791–32526250). The age-standardized DALYs rate (ASDR) decreased by 24%, dropping from 213.88 per 100 000 population (95% UI: 46.15–513.06) in 1990 to 163.37 per 100 000 population (95% UI: 32.53–379.07) in 2021, with an EAPC of −0.90 (95% CI: −0.96 to −0.84) (Table 2).

**TABLE 2:** Disability-adjusted life years (DALYs) and ASDR of IHD in 1990 and 2021, and temporal trends.

	1990		2021		1990–2021 EAPC of ASDR (95% CI)
	DALYs, No. ×10^3^	ASDR per 100,000 (95% UI)	DALYs, No. ×10^3^	ASDR per 100,000 (95% UI)	
Global	8296172 (1864218, 19526026)	213.88 (46.15, 513.06)	14079694 (2844791, 32526250)	163.37 (32.53, 379.07)	−0.90 (−0.96, −0.84)
**Socio-demographic index**					
High SDI	1704306 (241468, 4764930)	154.35 (21.89, 432.55)	1465436 (182101, 3865722)	69.85 (9.15, 182.14)	−2.68 (−2.76, −2.60)
High-middle SDI	2905619 (795310, 6280218)	299.85 (79.37, 656.18)	4192648 (1102473, 8865731)	212.74 (55.99, 453.24)	−1.30 (−1.49, −1.11)
Middle SDI	2372951 (669186, 4880342)	234.23 (61.74, 497.34)	5396748 (1305063, 11837145)	202.26 (46.34, 447.92)	−0.36 (−0.44, −0.28)
Low-middle SDI	1052899 (139931, 2754561)	169.69 (21.45, 451.79)	2491010 (255642, 6625092)	170.61 (16.56, 458.77)	0.16 (0.09, 0.22)
Low SDI	238626 (10148, 723979)	107.64 (4.57, 332.17)	516879 (18182, 1555614)	105.33 (3.49, 321.39)	0.03 (−0.04, 0.10)
**Region**					
Andean Latin America	20845 (840, 56454)	107.95 (4.37, 293.10)	37566 (1331, 104853)	64.70 (2.26, 180.56)	−1.90 (−2.32, −1.49)
Australasia	19314 (114, 80682)	83.79 (0.50, 348.05)	11303 (59, 45200)	21.94 (0.14, 85.53)	−4.56 (−4.73, −4.40)
Caribbean	36688 (319, 122637)	149.27 (1.35, 495.03)	47063 (290, 163477)	86.91 (0.54, 302.69)	−1.86 (−2.08, −1.64)
Central Asia	239712 (33880, 551013)	540.07 (73.76, 1246.48)	211942 (12891, 578864)	294.79 (17.69, 787.85)	−2.56 (−2.81, −2.31)
Central Europe	1137552 (409983, 2049664)	786.79 (278.81, 1422.38)	796007 (229498, 1523342)	351.02 (102.45, 672.80)	−2.96 (−3.09, −2.82)
Central Latin America	142454 (18166, 343710)	179.88 (22.20, 442.01)	343460 (38391, 879042)	138.86 (15.44, 355.87)	−1.06 (−1.27, −0.85)
Central Sub-Saharan Africa	9099 (1, 48511)	45.71 (0.01, 241.66)	21716 (7, 105538)	45.34 (0.02, 212.33)	−0.24 (−0.31, −0.16)
East Asia	2210912 (859519, 3873730)	263.36 (91.96, 474.55)	5480321 (1853877, 10283978)	256.49 (82.92, 493.16)	0.24 (0.01, 0.46)
Eastern Europe	829387 (55316, 2388780)	303.10 (20.12, 894.63)	833461 (48831, 2499021)	240.05 (15.60, 712.81)	−1.37 (−1.94, −0.79)
Eastern Sub-Saharan Africa	84212 (8141, 200864)	119.94 (11.39, 286.19)	142916 (7923, 381707)	95.39 (5.39, 249.54)	−1.04 (−1.14, −0.93)
High-income Asia Pacific	310577 (86532, 591412)	159.12 (42.84, 305.75)	223737 (29687, 508391)	46.71 (6.76, 105.64)	−4.32 (−4.49, −4.15)
High-income North America	372851 (1325, 1418139)	105.95 (0.40, 401.25)	453281 (12128, 1391778)	70.68 (2.13, 211.53)	−1.46 (−1.62, −1.30)
North Africa and Middle East	141771 (92, 698450)	81.84 (0.04, 412.62)	264553 (209, 1308962)	56.62 (0.03, 289.39)	−1.30 (−1.35, −1.26)
Oceania	7050 (955, 18059)	281.40 (38.95, 680.95)	18724 (2000, 47464)	277.79 (31.65, 674.07)	−0.19 (−0.31, −0.07)
South Asia	945664 (44334, 2717525)	155.08 (6.35, 451.59)	2790827 (190676, 7680610)	182.60 (11.53, 509.29)	0.81 (0.69, 0.92)
Southeast Asia	833522 (250063, 1603900)	326.26 (92.83, 635.59)	1621929 (324906, 3374912)	245.93 (47.14, 516.23)	−1.08 (−1.14, −1.02)
Southern Latin America	75842 (2430, 212306)	169.49 (5.25, 472.53)	58511 (1907, 160270)	66.68 (2.22, 183.06)	−2.74 (−2.89, −2.60)
Southern Sub-Saharan Africa	12915 (57, 46141)	46.01 (0.17, 169.63)	21729 (33, 89524)	37.74 (0.05, 160.50)	−0.69 (−1.03, −0.34)
Tropical Latin America	170229 (10184, 437729)	191.70 (11.15, 493.53)	231583 (11173, 634885)	89.51 (4.33, 246.02)	−2.48 (−2.56, −2.39)
Western Europe	645945 (17569, 2133211)	112.95 (3.41, 370.99)	350828 (9390, 1136354)	37.41 (1.27, 117.73)	−3.68 (−3.81, −3.54)
Western Sub-Saharan Africa	49631 (84, 193291)	60.85 (0.10, 237.26)	118237 (339, 422440)	66.54 (0.18, 241.49)	0.41 (0.27, 0.55)

ASDR, age-standardized DALYs rate; EAPC, estimated annual percentage change; CI, confidence interval; SDI, socio-demographic index; UI, uncertainty interval.

### The burden of ischemic heart disease associated with diet high in sodium at national level

**FIGURE 1:**
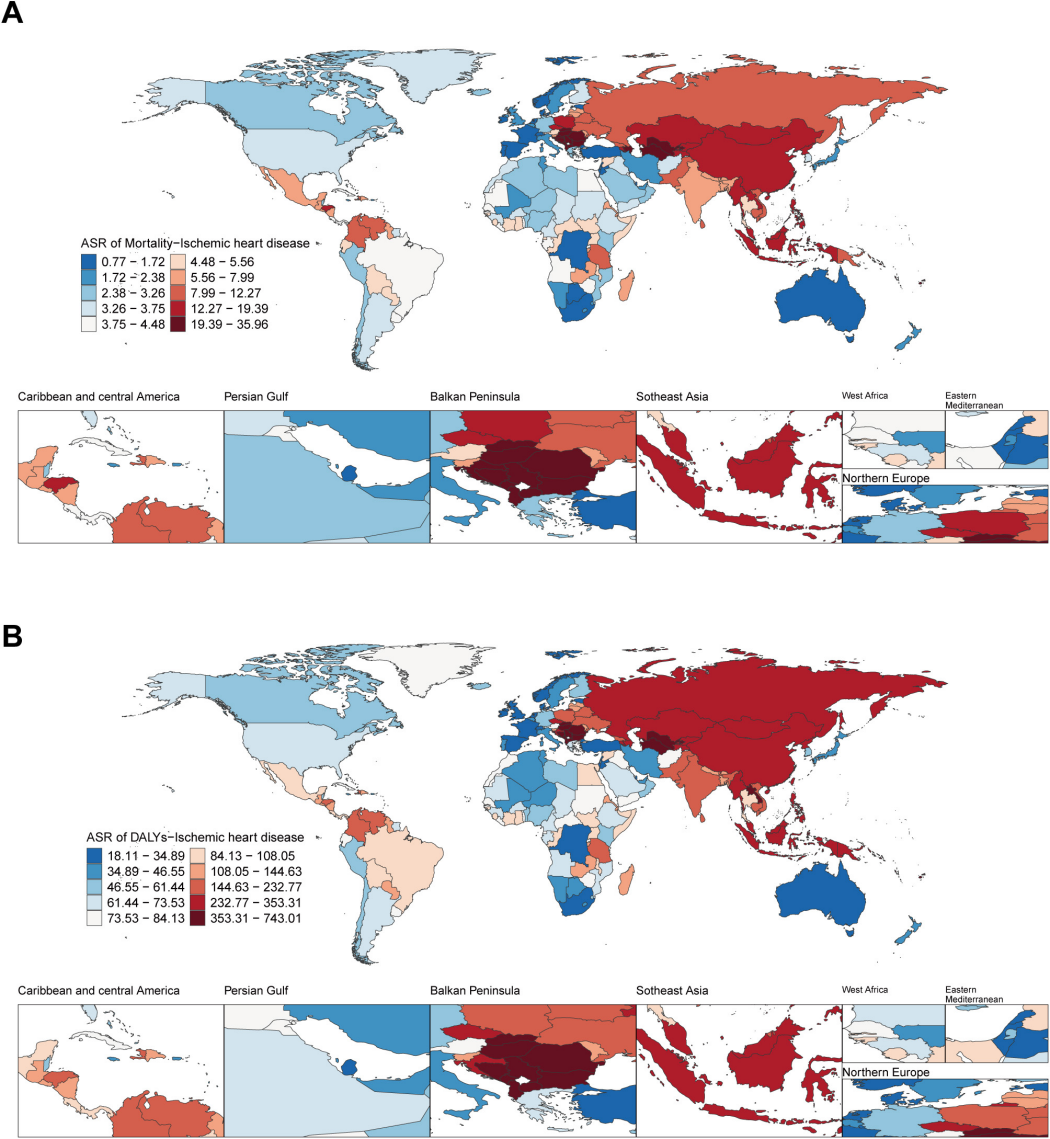
Global disease burden of ischemic heart disease associated with diet high in sodium in 204 countries and territories. **(A)** ASMR of ischemic heart disease in 2021; **(B)** ASDR of ischemic heart disease in 2021.

In 2021, at the national level, Nauru had the highest ASMR (35.60 per 100 000 population; 95% UI: 3.66–286.87), followed by Montenegro (32.63 per 100 000 population; 95% UI: 9.19–61.84) and Bulgaria (30.76 per 100 000 population; 95% UI: 8.67–58.33). Spain had the lowest ASMR (0.78 per 100 000 population; 95% UI: 0.01–3.21), closely followed by Australia (0.89 per 100 000 population; 95% UI: 0.002–4.02) and San Marino (0.92 per 100 000 population; 95% UI: 0.007–3.21). In 2021, Nauru and Bulgaria had the highest ASDR (18.29 per 100 000 population, 95% UI: 0.08–75.28; 18.41 per 100 000 population, 95% UI: 0.21–59.99) (Figures 1A, B).

**FIGURE 2:**
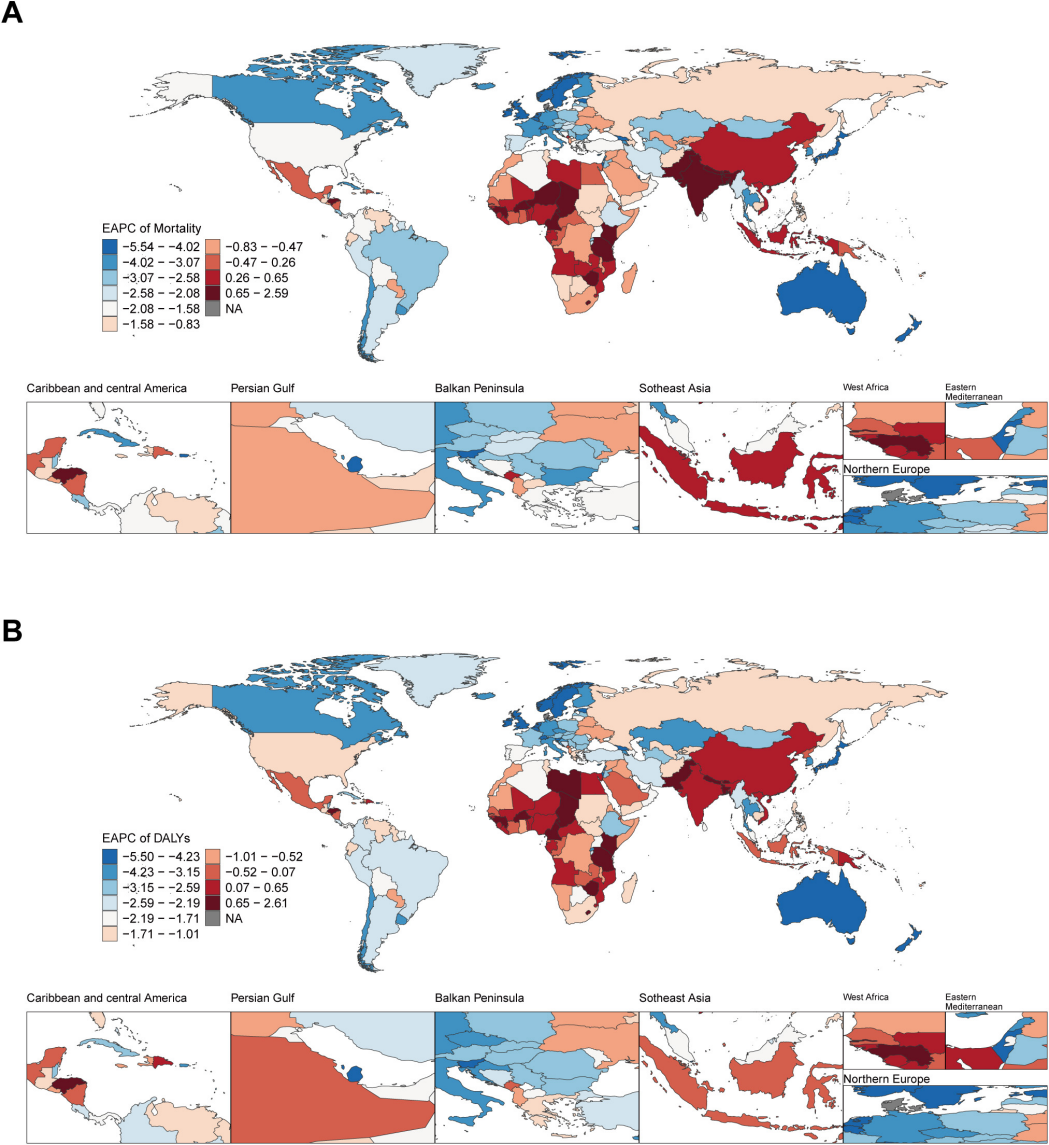
Global temporal trend of ischemic heart disease burden associated with diet high in sodium in 204 countries and territories from 1990 to 2021. **(A)** EPAC of the ASMR in ischemic heart disease; **(B)** EPAC of the ASDR in ischemic heart disease. EAPC, estimated annual percentage change.

From 1990 to 2021, at the national level, ASMR and ASDR showed a downward trend in most countries and territories. Pakistan had the highest growth rate of ASMR (EAPC: 2.56; 95% CI: 2.35–2.78), closely followed by Lesotho (EAPC: 2.36; 95% CI: 1.87–2.86) and Nepal (EAPC: 2.24; 95% CI: 1.93–2.54). Denmark experienced the fastest decline in ASMR (EAPC: −5.59; 95% CI: −5.79 to −5.40), followed by Israel (EAPC: −5.42; 95% CI: −5.64 to −5.19). From 1990 to 2021, Pakistan also had the highest growth rate of ASDR (EAPC: 2.58; 95% CI: 2.36–2.81), closely followed by Lesotho (EAPC: 2.56; 95% CI: 2.05–3.07). The most rapid decline in ASDR was observed in Denmark (EAPC: −5.56; 95% CI: −5.78 to −5.33), Norway (EAPC: −5.40; 95% CI: −5.50 to −5.30), and Israel (EAPC: −5.36; 95% CI: −5.62 to −5.11) (Figures 2A, B).

### The burden of ischemic heart disease associated with diet high in sodium at regional level

**FIGURE 3:**
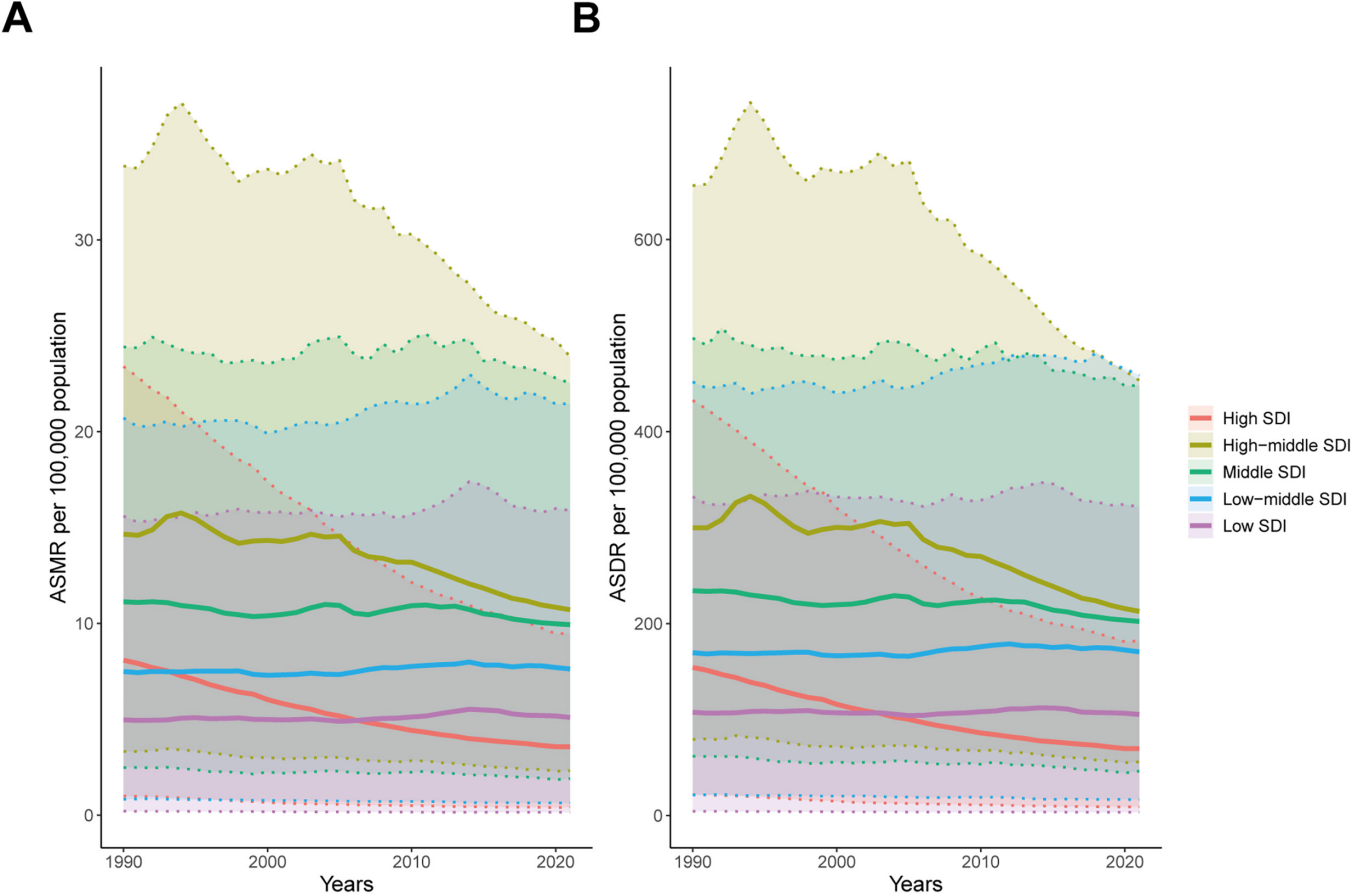
Age-standardized rates of ischemic heart disease associated with diet high in sodium at different SDI levels from 1990 to 2021. **(A)** ASMR; **(B)** ASDR.

From 1990 to 2021, high-middle SDI region had the highest ASMR and ASDR. In contrast, in 2021, high SDI region had the lowest ASMR and ASDR (Figures 3A, B and Tables 1, 2). The most rapid decline in both ASMR and ASDR was observed in high SDI region, followed by high-middle SDI region. In other regions, ASMR and ASDR remained largely unchanged (Figures 3A, B and Tables 1, 2).

**FIGURE 4:**
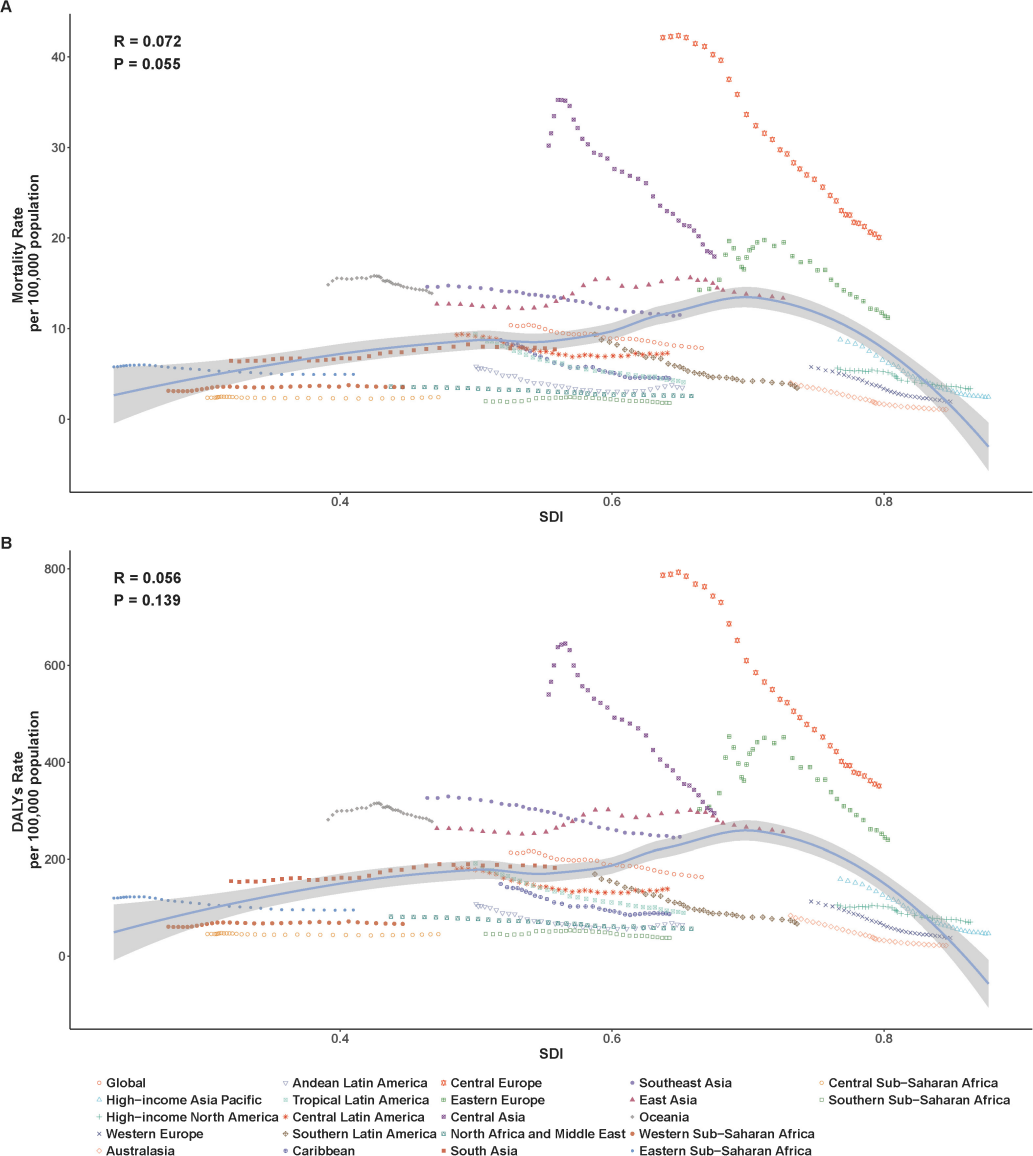
Age-standardized rates of ischemic heart disease associated with diet high in sodium among regions based on SDI levels from 1990 to 2021. **(A)** ASMR; **(B)** ASDR.

Subsequently, we examined the relationship between SDI and the corresponding ASMR and ASDR in 21 GBD regions from 1990 to 2021. At the regional level, ASMR and ASDR presented an inverted U-shaped relationship with SDI (Figures 4A, B). When the SDI was less than 0.7, ASMR and ASDR gradually increased as the SDI increased, and then decreased significantly. From 1990 to 2021, ASMR and ASDR showed a decreasing trend in most regions except South Asia, East Asia, and Western Sub-Saharan Africa (Figures 4A, B and Tables 1, 2). ASMR and ASDR in Central Europe and Central Asia were the highest (Tables 1, 2), exceeding the expected levels based on SDI from 1990 to 2021, but also showing significant declines (Figures 4A, B). Australia, the high-income Asia-Pacific region, and Western Europe had the fastest decline rates in ASMR and ASDR (Tables 1, 2).

### Age and sex patterns

**FIGURE 5:**
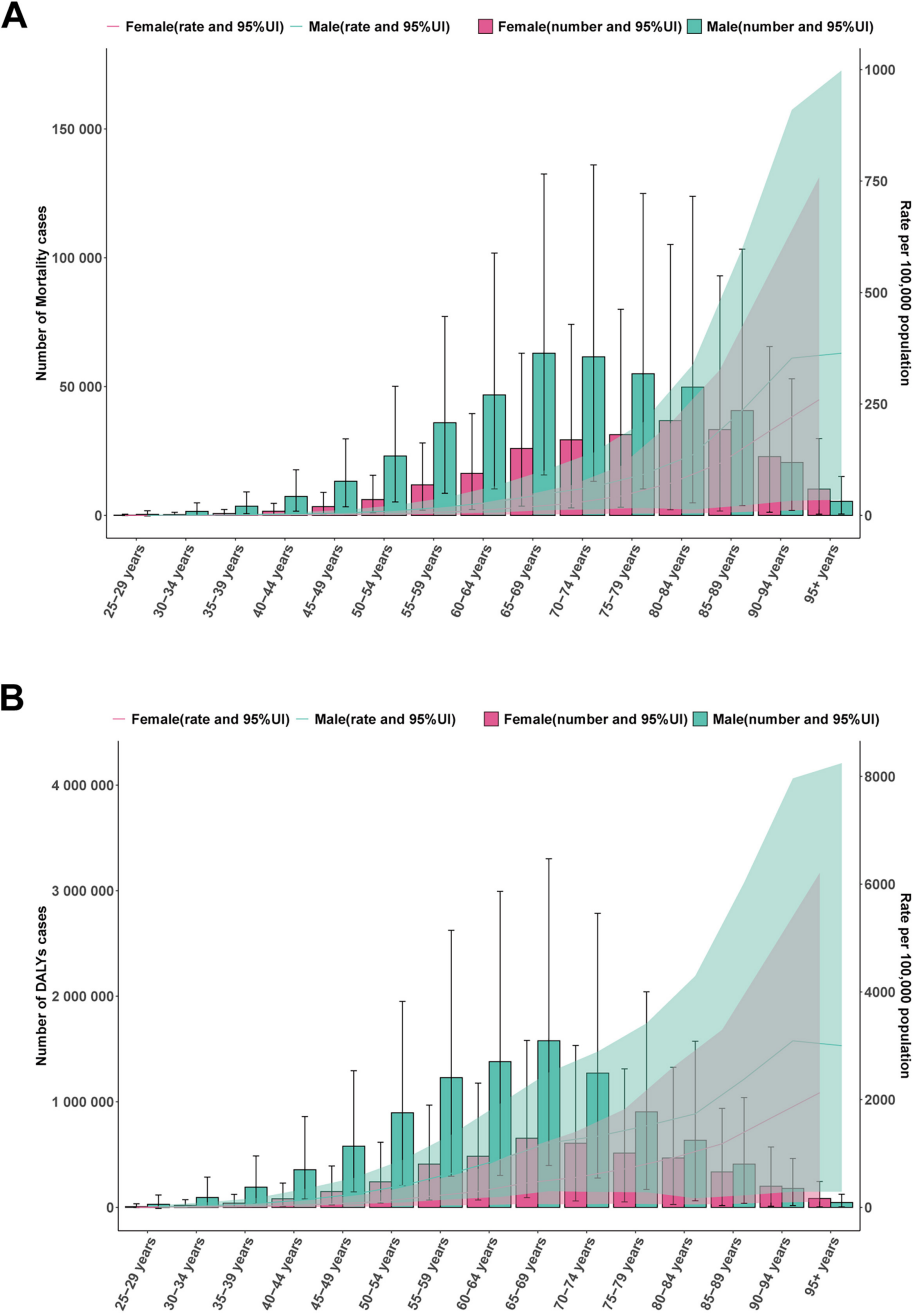
Global disease burden of ischemic heart disease associated with diet high in sodium in males and females by different age group in 2021. **(A)** Mortality cases and ASMR; **(B)** DALYs and ASDR.

**FIGURE 6:**
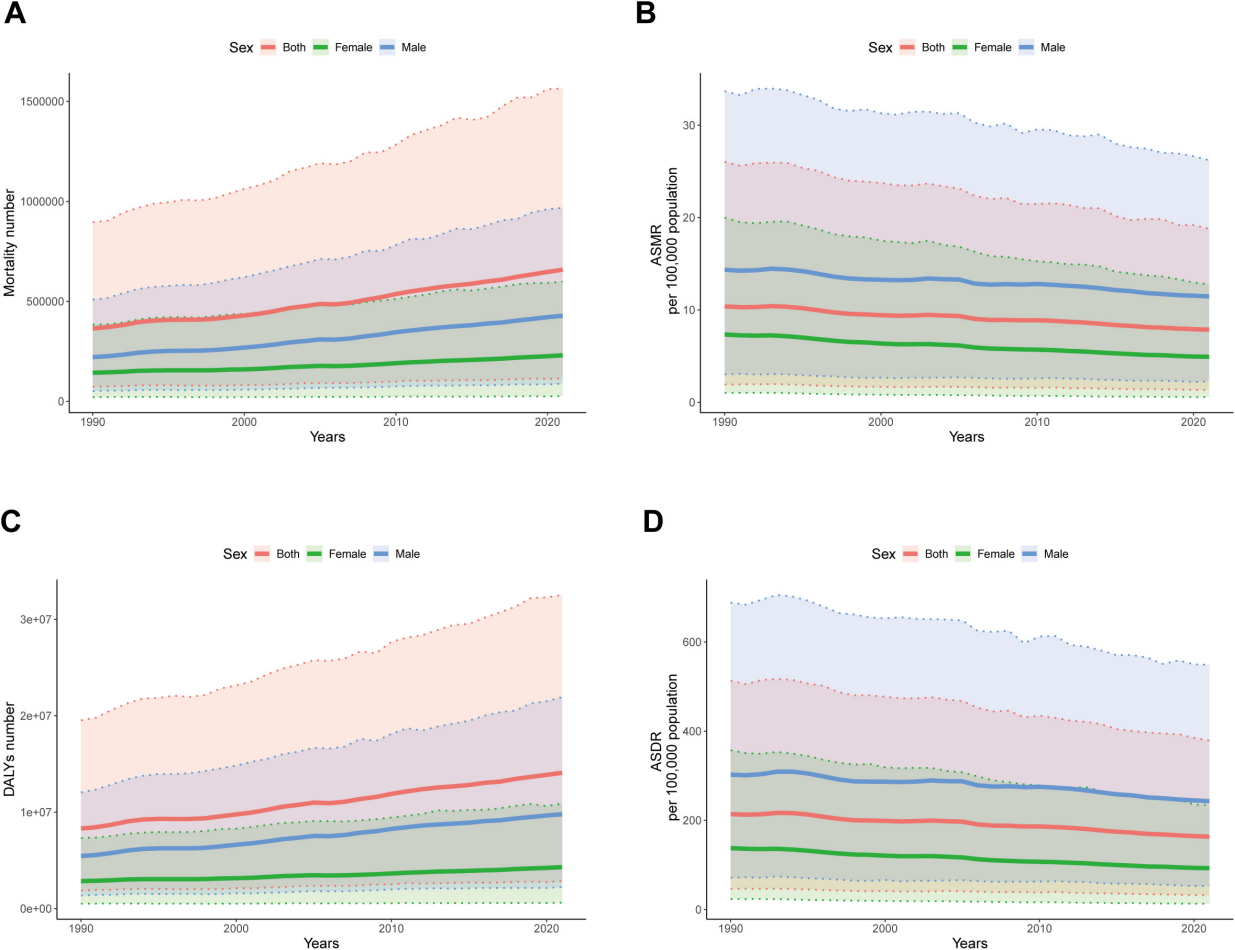
Global disease burden of ischemic heart disease associated with diet high in sodium with different sexes from 1990 to 2021. **(A)** Mortality number; **(B)** ASMR; **(C)** DALYs number; **(D)** ASDR.

In 2021, the number of mortality cases and DALYs of IHD associated with diet high in sodium were higher in males than in females before the age of 85–89. In males, the number of mortality cases and DALYs increased with age, reaching their peaks at 65–69 and 70–74 years, and then gradually declined with age. In females, the number of mortality cases attributed increased with age, reaching its peak between 80 and 84 years, and then gradually declined with age. ASMR and ASDR of males and females gradually increased with advancing age (Figures 5A, B). From 1990 to 2021, the number of mortality cases and DALYs for both males and females showed a continuous increasing trend, with males having a higher number than females (Figures 6A, C). Meanwhile, both ASMR and ASDR decreased, with males having significantly higher rates than females (Figures 6B, D).

**FIGURE 7:**
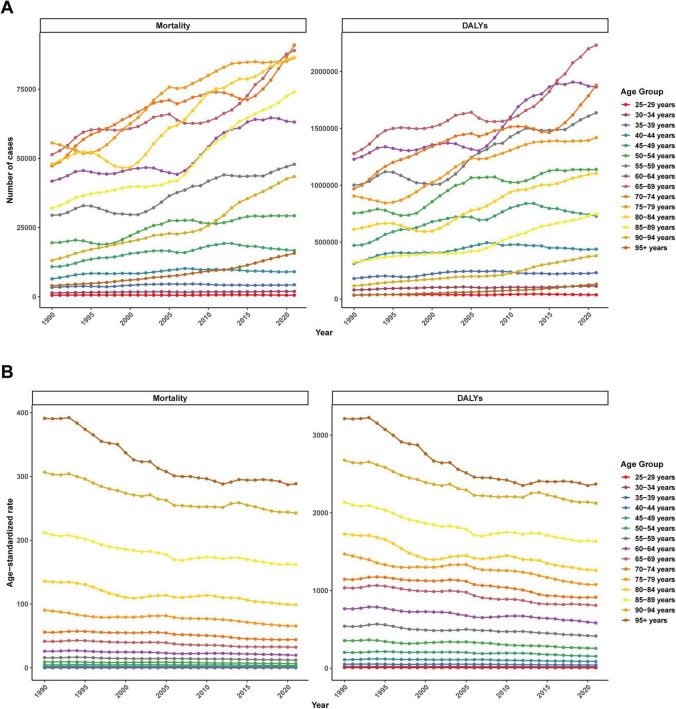
Global disease burden of ischemic heart disease associated with diet high in sodium by different age group from 1990 to 2021. **(A)** Mortality number and DALYs number; **(B)** ASMR and ASDR.

From 1990 to 2021, the number of mortality cases and DALYs in all age groups showed an overall increasing trend (Figure 7A). In contrast, ASMR and ASDR showed a decreasing trend in all age groups. The older the age, the higher the ASDR and ASMR (Figure 7B).

### Projection of the burden of ischemic heart disease associated with diet high in sodium

**FIGURE 8:**
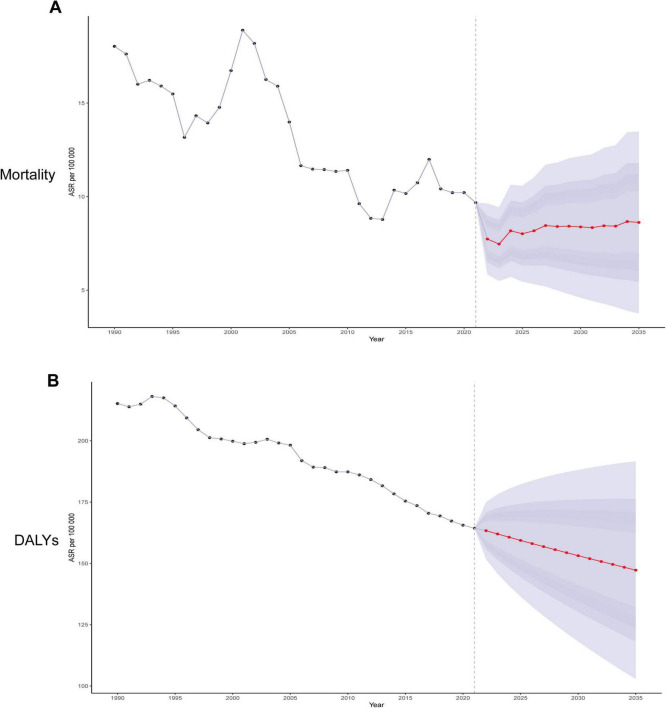
Trends in ischemic heart disease attributable to diet high in sodium with ASMR and ASDR in the global from 1990 to 2035 (BAPC models). **(A)** Trends in ASMR; **(B)** Trends in ASDR.

To clarify the development trend of IHD associated with diet high in sodium after 2021, we used the Bayesian age-period-cohort (BAPC) model to predict ASMR and ASDR from 2021 to 2035 (Figure 8). The BAPC model uses Bayesian methods to decompose the disease development trend into age, period, and cohort effects to quantify their independent impacts. The model projects the future disease burden (e.g., incidence) by extrapolating historical parameters, relying on stable age/cohort effects and continued period trends. The projection results indicate that from 2021 to 2035, ASDR will continue to decline, while changes in ASMR are not significant (Figures 8A, B).

## Discussion

This study conducted a comprehensive analysis of the global, national, and regional burdens of IHD associated with diet high in sodium from 1990 to 2021 and forecasted the development trend from 2021 to 2035. The results indicated that between 1990 and 2021, the number of mortality cases and DALYs associated with high-sodium diet as a risk factor for IHD increased globally, whereas the ASMR and ASDR exhibited a decreasing trend. However, the trends of ASMR and ASDR in SDI regions differed, and there were disparities in disease burden across different regions and countries. Comparisons based on age and sex revealed that the disease burden was higher among males than females, and the elderly population had the heaviest burden. The recent implementation guidelines of the WHO stress that population-level intervention measures, such as mandatory sodium content targets for processed foods, front-of-pack labeling, and mass media campaigns, are effective strategies for reducing sodium intake and preventing CVD (WHO ELENA Sodium-CVD Intervention, 2023). Consequently, controlling high-sodium intake is critical for mitigating mortality and DALY burden associated with IHD.

Sodium is an essential dietary mineral and electrolyte in human physiology, playing an indispensable role in sustaining life processes. It plays a pivotal role in supporting cellular homeostasis, facilitating neurotransmission, regulating fluid and acid-base equilibrium, and preserving plasma volume (15). However, high-sodium diet has emerged as a significant trigger for numerous non-communicable diseases. Diseases such as IHD, hypertension, and stroke are strongly associated with excessive salt intake. In fact, it has become the leading risk factor for diet-related deaths globally (16). Multiple studies have demonstrated a positive correlation between high dietary sodium intake and the onset or progression of IHD (17, 18). Studies indicate that each 1 g/day increase in sodium intake will elevate the risk of CVD by 6%, whereas reducing sodium intake may decrease the relative risk of atherosclerotic cardiovascular disease by approximately 9% (19, 20). In a high-sodium environment, the equilibrium of vascular endothelial cells is disrupted. This disruption causes abnormal vasoconstriction and accelerates atherosclerotic plaque formation (21). High-sodium diet can stimulate the production of reactive oxygen species (ROS) in vascular walls. Accumulated ROS trigger oxidative damage to endothelial cells, impairing the endothelial layer of blood vessels. Simultaneously, this process activates immune pathways (e.g., nuclear factor kappa B signaling), stimulates cytokine production (including IL-6), and aggravates vascular wall inflammation (21, 22). Excessive sodium intake results in an increase in extracellular fluid volume. When kidney function is compromised, this may give rise to the development of hypertension. Meanwhile, a decline in the reactivity of the renin-angiotensin-aldosterone system may enhance the salt-sensitivity of blood pressure (23, 24). Our study validated that high-sodium intake is a risk factor for IHD, which is further corroborated by earlier studies. A stratified analysis of disease burden statistics across different age groups, sexes, and geographic regions offers more direct and comprehensive evidence for clarifying the risk of IHD associated with elevated sodium intake and the necessity of dietary sodium reduction strategies.

This study revealed the persistent global burden of high-sodium-related IHD from 1990 to 2021. Despite a decreasing trend in ASMR and ASDR, there was a significant increase in absolute deaths and DALYs, which indicated that public health challenges remained unaddressed. This contradictory phenomenon can be attributed to population aging, the uneven distribution of medical resources, partial improvements in dietary sodium intake patterns, and regional differences in the implementation of sodium-reduction interventions. Our analysis revealed significant differences in the burden of IHD across different countries and territories. The disparities among these countries may stem from changes in dietary structure and the influence of public health policies. Countries with a high burden of IHD, such as Nauru and Bulgaria, often rely on high-sodium processed foods (such as cured meats and canned goods) or traditional high-salt diets. Coupled with inadequate regulation of sodium content in the food processing industry, limited coverage of health education, and scarce medical resources, these factors collectively exacerbate the harm of excessive sodium intake to the heart (15). Furthermore, middle-to-high SDI regions bore a heavier burden compared to high-SDI regions, as ASMR and ASDR exhibited an inverse U-shaped relationship with the SDI. These findings indicate a strong association among the level of socioeconomic development, the prevention and control of IHD, and salt-reduction strategies. High-SDI regions managed to significantly reduce the burden of sodium-related IHD burden through integrated systems of social welfare, universal healthcare, health literacy improvement programs, and proactive preventive measures. By 2021, these efforts led to the lowest regional ASMR and ASDR. This suggests that global sodium-reduction measures may have yielded relatively favorable outcomes. In 2019, reports indicated that 96 countries had implemented national salt-reduction initiatives, and an additional 16 countries were in the planning phase (25). The primary implementation strategies center on intervention measures in specific settings, food reformulation, consumer education, front-of-pack labeling, and salt taxes. Continuous monitoring and reporting of the progress of each intervention measure can assist in evaluating the effectiveness of policies, identifying gaps, and formulating follow-up measures required to achieve the goal of reducing salt intake by 30% (26). Conversely, middle-to-high SDI regions such as Central Europe and Central Asia, despite showing declining trends, still reported the highest SDI-related rate globally, which might be attributed to policy lag during economic transitions. The most concerning issue is that there was no significant decline in the ASMR and ASDR in South Asia, East Asia, and Sub-Saharan West Africa, suggesting synergistic effects of metabolic comorbidities (particularly elevated fasting blood glucose) and excessive sodium intake. Overall, these findings recommend the adoption of tailored prevention strategies, fully taking into account regional socioeconomic development trajectories, cultural dietary habits, and healthcare capacities. Such precision approaches are essential to disrupt the persistent global burden of sodium-related CVD.

Global epidemiological analyses demonstrate significant sex disparities in sodium-related cardiovascular pathology. In comparison to females, males shoulder a disproportionately higher burden of IHD associated with excessive sodium intake. From 1990 to 2021, age-adjusted mortality rates and DALYs were consistently higher among males than females, a trend corroborated by multiple longitudinal studies (27). Another crucial factor is that females can more effectively regulate the sodium balance in their bodies, rendering them less susceptible to the adverse effects of excessive sodium intake (28). Additionally, compared with females, males generally exhibit poorer dietary patterns and lifestyle habits, especially in aspects such as smoking and alcohol abuse (29, 30). Notably, it is reported that estrogen can offer vascular protection to females and impede the process of atherosclerosis (31). Therefore, this clinically significant sex difference demands greater attention to implement sex-specific dietary salt control measures. We also observed that the ASMR and ASDR for both males and females gradually increased with age, suggesting that older adults confronted a heavier burden of IHD due to high-sodium intake compared to younger populations. This phenomenon may be attributed to the following factors. Firstly, aging is associated with reduced vascular compliance and diminished renal filtration function, which may render older adults more vulnerable to the adverse effects of excessive sodium intake. Secondly, most elderly individuals already have other cardiovascular risk factors, such as arteriosclerosis and diabetes. High-sodium intake can exacerbate these pre-existing diseases, leading to more severe IHD (32). Additionally, due to diminished taste perception or lack of awareness of healthy eating habits, elderly individuals may increase their salt intake, resulting in long-term high-sodium intake issues (33). To enhance the quality of life of the elderly and alleviate the global disease burden, attention should be paid to their dietary habits while implementing strict sodium restriction strategies.

Nevertheless, this study still has certain limitations. Firstly, dependence on secondary GBD data rather than primary sources may limit reliability to some extent, which is a common limitation in previous GBD studies (34, 35). Secondly, inadequate understanding of asymptomatic IHD cases may lead to an underestimation of the burden of IHD associated with high-sodium intake. Underestimating the disease burden can influence policy-making, potentially leading to insufficient resource allocation and unnoticed financial burdens. Finally, when evaluating the impact of high-sodium intake on IHD using ASMR and ASDR, this analysis did not fully account for the severity of the disease.

## Conclusion

Our study demonstrated that high-sodium diet exerted a significant influence on the global burden of IHD. Meanwhile, it revealed substantial disparities in SDI regions, countries, age groups, and sexes. Utilizing the BAPC model, we analyzed the development trend of IHD associated with diet high in sodium from age, period, and cohort perspectives. Additionally, we were capable of predicting future changes and the disease burden of IHD. The prediction results of the BAPC model suggested that by 2035, the ASDR will continue to decline, whereas the change in the ASMR will be relatively minor. This implies that although current preventive strategies sustain the reduction of IHD mortality, control of disease burden has reached a plateau requiring breakthroughs through targeted interventions and integrated management. Therefore, priority should be placed on strengthening sodium-reduction interventions, particularly through targeted low-sodium diet education and evidence-based salt restriction policies, to mitigate the escalating burden of IHD globally.

## Data Availability

The original contributions presented in the study are publicly available. This data can be found here: Global Health Data Exchange query tool, http://ghdx.healthdata.org/gbd-results-tool.

## Footnotes

1
http://ghdx.healthdata.org/gbd-results-tool
